# Bronchoscopic retrieval of a bullet using a Dormia basket: a case report

**DOI:** 10.1186/1752-1947-8-358

**Published:** 2014-11-03

**Authors:** Elizabeth A Lax, Sayee H Kiran, Michael W Lee

**Affiliations:** 1 Department of Surgery, Providence Hospital and Medical Centers, 10061 West Nine Mile Road, Southfield, MI 48075, USA

**Keywords:** Bronchoscopic foreign body retrieval, Dormia basket, Penetrating neck trauma

## Abstract

**Introduction:**

Penetrating bullet injury to the trachea is a rarity, and therefore standardized procedure for injury management and foreign body removal has not been established. This is a case report describing retrieval of a bullet from the bronchus intermedius using flexible bronchoscopy and a Dormia basket.

**Case presentation:**

A 19-year-old African American woman presented with a gunshot wound to her right neck just lateral to the sternal head of the sternocleidomastoid muscle and just superior to the clavicle. Imaging showed a bullet lodged within her right bronchus with no evidence of vascular injury. Fiberoptic flexible bronchoscopy in combination with biopsy forceps and a Dormia basket were used for bullet removal.

**Conclusions:**

Removal of foreign bodies from the airway is essential in order to avoid complications such as tracheal stenosis, pneumonia, bronchiectasis and foreign body migration. To the best of our knowledge, this case is one of only several cases reporting the use of flexible bronchoscopy for bullet removal, and this is the only case to describe the use of a Dormia basket. This case demonstrates bronchoscopy as a safe and effective means of minimally invasive removal of a bullet fragment from the bronchus in a trauma setting.

## Introduction

Tracheal injury secondary to penetrating trauma is a rare occurrence. However, if this injury goes unrecognized it can lead to serious sequelae which can become life-threatening. A retained foreign body within the trachea or bronchus can lead to the development of airway strictures, as well as atelectasis, infection, and perforation. Given the rarity of this injury, a standard procedure for the retrieval of foreign bodies within the airway in a trauma setting has yet to be established. There have been only a few case reports describing the successful management of retained ballistic fragments within the airway using a bronchoscopic technique [1-4]. Here we report a case of successful retrieval of a bullet from the right bronchus intermedius using flexible fiberoptic bronchoscopy (FOB) and a Dormia basket.

## Case presentation

**Figure 1:**
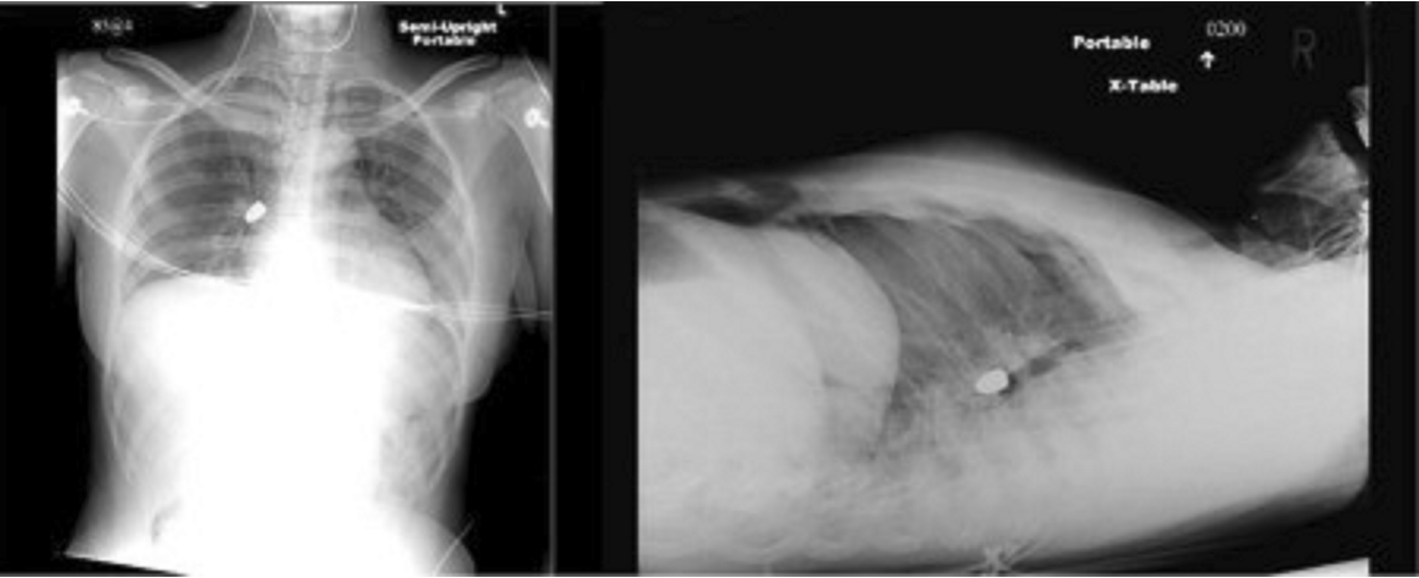
Chest X-ray showing a bullet located within right paracardiac position.

**Figure 2:**
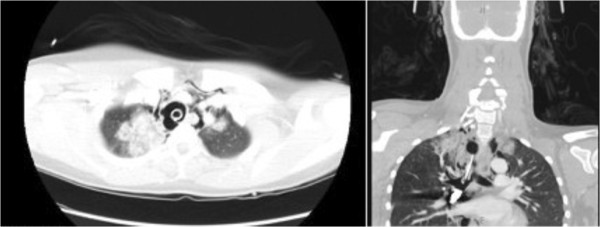
Computed tomography scan showing a posterior tracheal defect, and a bullet within the right bronchus.

**Figure 3:**
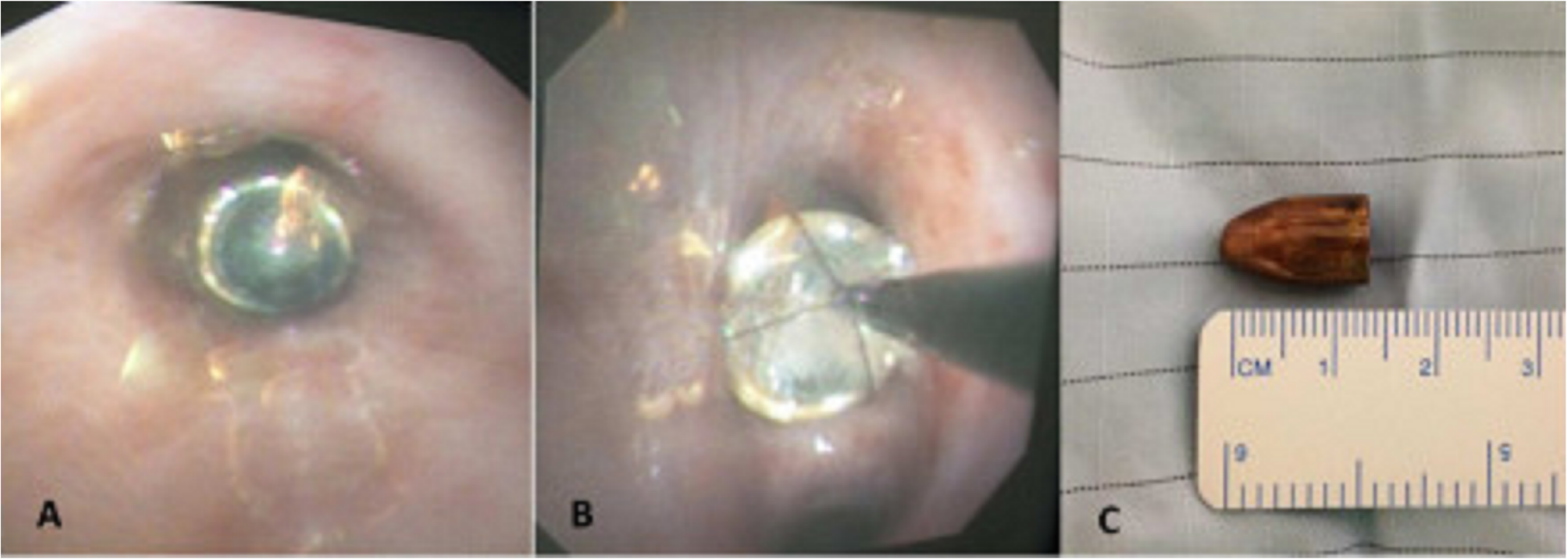
Images of bullet A-B Bullet seen within the right bronchus intermedius during bronchoscopy, C Bullet retrieved from bronchus using a Dormia basket.

**Figure 4:**
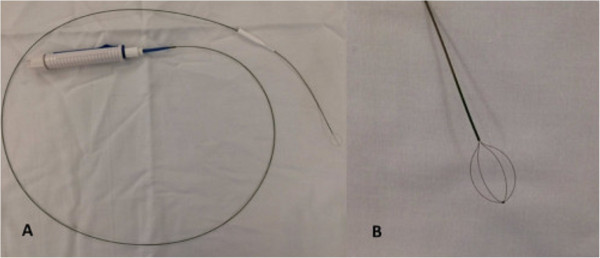
A-B Dormia basket.

A 19-year-old African American woman sustained a gunshot wound to her neck. On initial evaluation, approximately 20 minutes after injury, she was alert, oriented, speaking full sentences and was complaining of pain to her neck. An examination revealed a gunshot wound to her right neck just lateral to the sternal head of the sternocleidomastoid muscle and just superior to the clavicle. The wound was non-bleeding and air was escaping. No other wounds were noted. Initial vital signs were heart rate 118, blood pressure 144/85mmHg, respiratory rate 20 breaths/minute, pulse oximetry 100% on 15L non-rebreather, breath sounds were present bilaterally, and there was no evidence of subcutaneous emphysema. A chest X-ray showed a bullet overlying her medial right midlung field and superimposing the right hilum, and also smaller fragments in the right apex, as well as pneumomediastinum (Figure 1). She was intubated for airway protection. Computed tomography (CT) with intravenous contrast showed significant pneumomediastinum extending to her skull base, a bullet lodged within the right bronchus intermedius, and a luminal irregularity of the distal trachea. There was no evidence of vascular injury on CT (Figure 2).She was taken to the operating room. Flexible FOB was performed. The bullet was visualized within the right bronchus intermedius (Figure 3A). Retrieval of the bullet was attempted with a combination of biopsy forceps and a Dormia basket. The bullet could be moved but not extracted using biopsy forceps. Next a Dormia basket was advanced passed the bullet, opened, and closed around the bullet (Figure 3B-C, Figure 4). The basket with bullet ensnared and endotracheal tube were removed together. She was reintubated once the bullet was confirmed to have been retrieved. Postretrieval bronchoscopy was performed and a tracheal injury was visualized on the posterior trachea. An esophagogastroduodenoscopy was preformed which did not reveal any injury to her esophagus.

Next, a neck exploration via a collar incision was performed. The trajectory of the bullet was between her common carotid artery and right subclavian artery. Injury to the cervical portion of her posterolateral trachea and membranous disruption of proximal intrathoracic trachea was identified. Primary repair of the tracheal injury was performed. She tolerated the procedure well, and was transported to our Intensive Care Unit (ICU) intubated postoperation. She was extubated 2 days after surgery and was subsequently discharged home without complications.

## Discussion

Tracheal injury after penetrating trauma is a rare event and has been reported to occur in less than 1% of penetrating traumas to the chest [5]. The urgency to identify and treat tracheal injuries is well documented; however a standardized technique for removal of foreign bodies during an emergency situation is not established. The most common signs of airway injury are subcutaneous emphysema pneumothorax and hemoptysis [6,7]. A pathognomonic sign of airway laceration is air escaping from a penetrating wound in the neck [7-10]. A high level of suspicion and liberal use of bronchoscopy are necessary for early identification and treatment of airway injuries [6].

In general, FOB is used for evaluation of distal airways and basic procedures such as biopsy, bronchoalveolar lavage, and suctioning of mucus plugs and blood clots. Complications of FOB include airway obstruction, hypoxemia, and mechanical trauma to the trachea and bronchus. In the event of airway obstruction or failure to remove a foreign body while using FOB, it is important that a rigid bronchoscope be readily accessible. The rigid scope allows for better airway control with the administration of ventilatory support via a side port, and a larger array of instruments such as forceps, hooks and balloons, to choose from [9].

A Dormia basket is an endoscopic retrieval device developed in 1961 by an Italian Urologist, Enrico Dormia. The original design consisted of a four-wire basket used for ureteral stone retrieval. Modified versions now incorporate three, four or six stainless steel wire baskets in flat or helical arrangement with a range of wire stiffness and basket width options. These modifications have allowed for expanded application of the device for use during extraction of biliary stones, polyps, food bolus impactions, and foreign body aspirations [11]. FOB is particularly useful in the pediatric population where foreign body aspiration is common. The pediatric literature has reported FOB as a safe and useful means of foreign body retrieval generally with biopsy or grasping forceps [7,9].

The use of FOB has been documented for removal of foreign bodies secondary to aspiration [7,9]. Previous reports of retained bullets within the airway have also employed bronchoscopy for retrieval [1-4]. O’Connor *et al*. report a case of retained bullet removal from the left lower lobe bronchus. Fulginiti *et al*. described the removal of a bullet using flexible FOB and biopsy forceps in a 23-year-old patient in the ICU setting who was mechanically ventilated. Kiev *et al*. report bronchoscopic bullet removal 9-years postinjury. Choh and Adler describe the use of rigid bronchoscopy and forceps for removal of a retained bullet in the right lower lobe bronchus. This report to the best of our knowledge is the first to describe such usage of a Dormia basket for the purpose of retained bullet removal from the airway.

## Conclusions

Removal of foreign bodies from the airway is essential in order to avoid complications such as tracheal stenosis, pneumonia, bronchiectasis, and bullet migration. This case emphasizes the necessity to quickly evaluate the airway in cases of penetrating injury to the neck, and demonstrates FOB and the use of a Dormia basket as a safe and effective means of minimally invasive removal of a bullet fragment from the bronchus during an emergency setting.

## Consent

Written informed consent was obtained from the patient for publication of this case report and accompanying images. A copy of the written consent is available for review by the Editor-in-Chief of this journal.

## Abbreviations

CT: Computed tomography; FOB: Fiberoptic bronchoscopy; ICU: Intensive Care Unit.

## Competing interests

The authors declare that they have no competing interests.

## Authors’ contributions

Concept and design: EL, SK, and ML. Data acquisition/literature review: EL and SK. Drafting of manuscript: EL, SK, and ML. Critical revision: ML. Final approval of submission: EL, SK, and ML. All authors read and approved the final manuscript.
